# Covariates of high-risk sexual behaviour of men aged 50 years and above in sub-Saharan Africa

**DOI:** 10.1080/17290376.2017.1392340

**Published:** 2017-10-26

**Authors:** Clifford O Odimegwu, Nyasha Mutanda

**Affiliations:** ^a^ Demography and Population Studies Programme, Schools of Public Health and Social Sciences, University of the Witwatersrand, Johannesburg, South Africa, *Email: nchadoka@gmail.com

**Keywords:** sexual behaviour, older men, sub-Saharan Africa, comportement sexuel, hommes âgés, Afrique subsaharienne

## Abstract

Since the advent of HIV/AIDS, sexuality studies in sub-Saharan Africa (SSA) have focused mainly on the sexual behaviour of the younger generation (15–49 years) and little has been done to understand the sexual behaviour of those a 50 years and above. The objective of this study is therefore to examine the covariates of high-risk sexual behaviour among men aged 50 years plus within the SSA region. Data from Demographic and Health Surveys of 10 SSA countries were pooled together and a sample of 5394 men aged 50 years plus who have ever had sex was analysed. Findings show that in SSA, a large proportion of men aged 50 years plus (74%) were sexually active and a substantial proportion of these men engaged in unsafe sexual behaviours, such as having multiple sexual partners and unprotected sex. The multivariate logistic regression analysis showed that involvement with multiple sexual partners was significantly associated with older age, urban residence, religion, having primary or secondary education, and ever taken an HIV test. Condom use at last sex was significantly associated with age at first sex, multiple sexual partners, level of education and ever been tested for HIV. These results suggest that HIV prevention and intervention programmes should also target older men as they are also sexually active and at risk of being infected because of unsafe sexual practices.

## Résumé

Depuis l'avènement du VIH / SIDA, les études sur la sexualité en Afrique subsaharienne (ASS) se sont concentrées principalement sur le comportement sexuel du groupe en âge de procréer (15-49 ans) et peu de choses ont été faites pour comprendre le comportement sexuel des 50 ans et plus. L'objectif de cette étude est donc d'examiner les covariables des comportements sexuels à haut risque chez les hommes âgés de 50 ans et plus dans la région d’ASS. Les données des Enquêtes Démographiques et de Santé de 10 pays d'Afrique subsaharienne ont été regroupées et un échantillon de 5 394 hommes âgés de 50 ans et plus ayant déjà eu des rapports sexuels a été analysé. Les résultats montrent qu'en Afrique subsaharienne, une proportion importante d'hommes âgés de 50 ans et plus (74%) étaient sexuellement actifs et qu'une proportion significative de ces hommes avait des comportements sexuels à risque, tels qu’avoir des partenaires sexuels multiples et des rapports sexuels non protégés. L'analyse de la régression logistique multivariée a montré que le fait d’avoir de multiples partenaires sexuels était significativement associé à un âge plus avancé, la résidence urbaine, la religion, au niveau d’enseignement primaire ou secondaire et au fait d’avoir déjà passé un examen de dépistage du VIH. L'utilisation du préservatif lors du dernier rapport sexuel était significativement associée à l'âge au premier rapport sexuel, aux multiples partenaires sexuels, au niveau de scolarité et au fait d’avoir déjà fait un test de dépistage du VIH. Ces résultats suggèrent que les programmes de prévention et d'intervention contre le VIH devraient également cibler les hommes âgés car ils sont également sexuellement actifs et risquent d'être infectés à cause de pratiques sexuelles à risque.

## Introduction

Since the emergence of HIV/AIDS in sub-Saharan Africa (SSA) the subject of human sexual behaviour has received considerable attention in scientific research. The target population of the majority of studies on sexual behaviour in SSA has been the younger generation, particularly those aged between 15 and 49 years. The older segment of the population, classified as older adults (50 years and above), has thus been neglected. This gap in knowledge has been attributed to various reasons. Freeman and Anglewicz (2012) argued that research on the sexual behaviour of the older adults in developing countries has been limited due to lack of large population-based data that captures the sexual behaviour of persons aged 50 years and above. Furthermore, Gott and Hinchliff (2003) pointed out methodological challenges that are faced when dealing with older people related to the sensitivity of the topic and validity of accounts of sexual behaviour and attitudes.

Despite the older adults (50 years plus) being excluded from research on HIV and sexual behaviour, existing evidence from a few studies conducted in SSA and several studies in other regions show that people remain sexually active beyond the age of 50 years (Araujo, Mohr, & McKinlay, 2004; Cooperman, Arnsten, & Klein, 2007; DeLamater & Sill, 2005; Golub et al., 2010; Knodel & Chayovan, 2001; Lindau et al., 2007; Lovejoy et al., 2008; Peltzer, Phaswana-Mafuya, Mzolo, Tabane, & Zuma, 2010; Todd et al., 2009). Todd et al. (2009), using longitudinal cohort data from Uganda, South Africa and Zimbabwe showed that 90% of men aged between 50 and 65 years were sexually active. In a related study using the 2005 South African HIV prevalence and behaviour survey, Peltzer et al. (2010) reported that about 41.1% of the older population aged 50 years and above were sexually active within the 12 months prior to the survey, with men being more sexually active compared to women of the same age group. However, sexual activity and desire tend to decline with increasing age among persons aged 50 years and above (Araujo et al., 2004; Call, Sprecher, & Schwartz, 1995; Knodel & Chayovan, 2001; Lindau et al., 2007; Marsiglio & Donnelly, 1991).

Age has also been found to be significantly associated with high-risk sexual behaviours and extramarital sex (Hill, Cleland, & Ali, 2004; Isiugo-Abanihe, 1994; Kimuna & Djamba, 2005; Oyediran, Isiugo-Abanihe, Feyisetan, & Ishola, 2010). Studies in SSA countries like Malawi, Nigeria, Uganda, Zambia and Zimbabwe have shown that high-risk sexual behaviours such as having multiple sexual partners and engaging in unprotected intercourse with non-spousal partners are common among older men (Freeman & Anglewicz, 2012; Kimuna & Djamba, 2005; Kinga, Issacb, Ademolab, & Nnennad, 2010; Todd et al., 2009). Having multiple concurrent sexual relationships, inconsistent and non-use of condoms are some of the major risk factors for sexual transmission of HIV infection within the region (Ahmed et al., 2001; Mah & Halperin, 2010; Morris & Kretzschmar, 1997).

Furthermore, high-risk sexual behaviour by the older adults can lead to increased prevalence of HIV/AIDS among the younger age groups. This is because other studies have found that some older men are involved in sexual relationships with younger women over whom they have power and influence regarding non-use of condoms (Kaufman & Stavrou, 2004; Leclerc-Madlala, 2008; Meekers & Calvès, 1997; Silberschmidt & Rasch, 2001).

An increase in the HIV-prevalence rate among older people aged 50 years and above has also been reported. Mahy, Autenrieth, Stanecki, and Wynd (2014) reported that the global HIV-prevalence rate among adults aged 50–54 years doubled between 1995 and 2013, reaching 0.51% in 2013. Approximately 2.5 million older adults aged 50 years and above in SSA were HIV-positive in 2013 (Mahy et al., 2014). Freeman and Anglewicz (2012) study in rural Malawi revealed that HIV prevalence among older men (50–64 years) was two times higher than that of men aged between 15 and 49 (8.9% compared to 4.1%, respectively). National surveys in South Africa reported that about 11% of people aged 50–54; 5% of those aged 55–59; and 4% among those aged 60+ reported HIV (Peltzer et al., 2010; Shisana et al., 2005).

Studies on sexual behaviour and its determinants among older men are therefore essential in SSA region. This is because understanding the determinants of high-risk sexual behaviour by older men provides a knowledge platform on which relevant interventions targeted at the men can be conceived. This study, therefore, examines the covariates of high-risk sexual behaviour among men aged 50 years and above in SSA.

## Data, variables and methods

### Data

This study is based on data from Demographic and Health Surveys (DHSs) that were conducted in 10 SSA countries in the mid-2000s as part of the DHS Programme (see Table 1). The selection of these countries was based on the availability of data on men aged 50 years and above in each survey because not all DHSs have included these men. DHSs are nationally representative surveys that collect detailed information on; demographic and socio-economic characteristics, sexual behaviour (recent sexual activity, number of sexual partners in the last 12 months prior to the survey, use of a condom during the most recent sexual act), knowledge of HIV/AIDS and perception on risk of HIV; among other things, from a nationally representative sample of women aged between 15 and 49 years and men aged between 15 and 59 years, – in certain surveys the upper limit for men is 64 years.

**Table 1.** Sample sizes from DHS in SSA region that were pooled together (weighted).

Country	DHS round	Men’s response rate	Sample size
Cameroon	2011	95.6	731
Kenya	2008	88.6	207
Lesotho	2009	95.0	293
Malawi	2010	92.2	354
Mozambique	2011	96.2	521
Nigeria	2008	92.6	1670
Rwanda	2011	98.7	635
Senegal	2010/2011	87.0	497
Uganda	2011	89.2	122
Zimbabwe	2010/2011	85.8	364
Total SSA sample			5394

Note: Response rate is for all eligible men who participated in survey.

Detailed information about the sampling techniques and data collection can be found in individual country reports accessed at http://www.measuredhs.com. Briefly, the sample for all the DHSs is selected using a stratified two-stage cluster design (ICF International, 2012). In the first stage, sampling clusters (usually census enumeration areas) are selected proportional to size, the size being the number of households in the cluster. In the second stage, a sample of households is selected from a complete household listing for the cluster. In all the DHSs three questionnaires (household, women and men) were used to collect information from eligible respondents (women aged between 15 and 49 years and men aged between 15 and 59/64 years) through face to face interviews. The questionnaire of interest for this study was the men’s questionnaire which was used to collect information from eligible men. Specifically, data on background characteristics and sexual behaviour that was collected from male respondents aged 50 years and above.

### Ethical consideration

The ethical approval for each of the selected DHS was obtained from the relevant national ethics committees and the Ethical Committee of ICF at Calverton, Maryland, USA prior to the survey, therefore, no further ethical approval was needed for this study.

### Specification and measurement of dependent variable

The dependent variable for this study, high-risk sexual behaviour, was measured in two forms, namely having multiple sexual partners and use of condom at last sex. Having multiple sexual partners was a binary outcome variable measured as one (1) if a man reported multiple sexual partners in the last 12 months prior to the survey and zero (0) if not. Condom use at last sex was also categorized as one (1) if a man reported that they used a condom during their last sex and zero (0) if they reported that they did not use a condom during their last sexual encounter. A focus on these two variables is key because having multiple concurrent sexual partners and unprotected sex are some of the major pathways through which HIV/AIDS spread within the SSA region (Ahmed et al., 2001; Mah & Halperin, 2010).

### Independent variables measurement and description

Independent variables that were selected for this study were; age, age at first sex, place of residence categorized as urban or rural, religion was divided into Catholics, other Christians, Muslims and other – the ‘other’ category includes those who reported affiliation to traditional religion and those who were not affiliated to any religion. Type of marriage was divided into two – monogamous marriage if a man reported that he is married to one wife and polygamous marriage if a man is married to more than one wife. The level of education was categorised as no education, primary education and secondary or above, wealth index is divided into poor, middle and rich. The wealth index variable in the DHS is a composite measure of household’s cumulative living standard, calculated using data on household’s ownership of assets such as television, type of water accessed, sanitation, materials used for housing construction among other things; visit www.dhsprogram.com/topics/wealth-index for more information. Further, ever been tested for HIV was coded as yes if a man had ever tested for HIV at one point in their lifetime and no if not. HIV/AIDS transmission and prevention knowledge was categorized as high, medium and low.

The HIV/AIDS transmission and prevention knowledge variable was created from respondents’ responses to questions on HIV/AIDS included in the DHS men’s questionnaire, such as can people; (i) reduce their chances of getting HIV by having just one sex partner who is not infected and who has no other partners? (ii) get HIV from mosquito bites? (iii) reduce their chances of getting HIV by using a condom every time they have sex? (iv) get HIV by sharing food with a person who has AIDS? (v) reduce their chance of getting HIV by abstaining from sexual intercourse? (vi) is there anything (else) a person can do to avoid or reduce the chances of getting HIV or AIDS? (vii) what can a person do? abstain from sex, use condoms, limit sex to one partner/stay faithful to one partner, limit number of sexual partners, (ICF International, 2011). Response options for each question were yes, no, don’t know. Responses were scored for the number of accurate responses (range 0–12). Correct responses were scored 1, incorrect and don’t know responses were scored 0.

### Statistical analysis

Survey data from individual countries were pooled together to form one dataset for SSA for the analysis. Pooling of datasets was done so that we could have a large sample size yielding meaningful statistical results.

Our analytical approach included univariate, bivariate and multivariate analysis. The univariate analysis shows the distribution of respondents by key variables. The bivariate logistic regression examines the bivariate relationship between each of the selected variables and sexual behaviour variables (condom use at last sex and multiple sexual partners). Finally, the multivariate logistic regression analysis examines the net effects of the variables on older men’s sexual behaviour. The first model examines the net effects of the selected independent variables on condom use during last sexual encounter while the second model examines the effect of the socio-economic and demographic variables on having multiple sexual partners. The level of significance for all statistical tests was 0.05. The pairwise correlation matrix was conducted to check for the strength of the linear relationship between variables and no variables were found to be highly correlated with each other.

Data were analysed using STATA version 12.0 (StataCorp. 2011). Sampling weights contained in the DHS dataset were used in the data analysis to correct for any differentials in response rate and for any unequal probability used to select the subject in the sample.

## Results

### Profile of the study population

The total sample for this study was 5394 men aged between 50 and 64 years. The average age of the respondents was 54 years and the average age at first intercourse was 20 years. Most of the respondents were rural residents (69%) and monogamy was the most dominant type of marriage among the older men (Table 2). About 45% of the respondents had a primary level of education and 42% had a wealth index of rich.

**Table 2.** Description of men aged 50+ in SSA by sexual behaviour, demographic, socio-economic and variables; Sample size 5394.

Variables	Frequency	%
*Current sexual activity*		
Active	3949	74.2
Not active	1445	25.8
*Extramarital partners*		
Yes	492	9.3
No	4902	90.7
*Condom use during last sex*		
Yes	318	6.5
No	5076	93.5
*HIV Prevention/transmission knowledge*		
Low	525	9.7
Medium	2214	41.1
High	2654	49.2
*Ever been tested for HIV*		
No	3442	65.7
Yes	1793	34.3
**Age**	Mean 54 years	
*Place of residence*		
Urban	1693	31.4
Rural	3701	68.6
*Religion*		
Catholics	1356	25.1
Other Christians	1868	34.6
Muslim	1647	30.5
Other	523	9.7
**Age at 1st Intercourse**	Mean = 20 years	
*Type of marriage*		
Monogamy	3872	77.6
Polygamy	1115	22.4
*Education level*		
No education	1680	31.1
Primary	2448	45.4
Secondary+	1265	23.5
*Wealth index*		
Poor	2022	37.5
Middle	1112	20.6
Rich	2260	41.9

Out of the 5394 men aged 50 years and above, about 74% of the respondents reported that they had been sexually active within four weeks prior to the survey and 9% of the older men reported that they had sexual encounters with multiple sexual partners (excluding spouse) in the past 12 months prior to the survey (Table 2). Over 90% of the men reported that they did not use a condom during their last sexual encounter. With regards to accurate knowledge of how HIV can be prevented or transmitted, 49% of the men had a score of high knowledge and 41% had medium knowledge. However, 66% of the men reported that they had never taken an HIV test in their lifetime.

### Results from the bivariate analysis

#### Condom use

Results in Table 3 show that compared to urban residents, rural residents were less likely to use a condom during last sex (unadjusted odds ratios (UOR) 0.50; confidence interval (CI) 0.53–0.85) and polygamists had lower odds of condom use at last sex compared to monogamists (UOR 0.45; CI 0.31–0.65). Muslim men (UOR 0.41; CI 0.29–0.58) had lower odds of condom use at last sex compared to Catholics. Men who had secondary or higher level of education (UOR 6.54; CI 4.35–9.83) were more likely to use a condom during last sex compared to those with no education. Men from households with a wealth index of middle (UOR 1.80; CI 1.27–2.59) and rich (UOR 2.77; CI 2.07–3.69) were more likely to report condom use at last sex compared to men from poor households. Men who had ever tested for HIV were more likely (UOR 3.26; CI 2.57–4.12) to use a condom at last sex compared to those who had never taken an HIV test. Men whose HIV transmission and prevention knowledge score was medium (UOR 3.45; CI 1.72–6.94) or high (UOR 4.33; CI 2.17–8.65) were more likely to have used a condom at last sex compared to those men whose HIV knowledge score was low. Those men who reported having multiple sexual partners (UOR 6.29; CI 4.89–8.09) were more likely to have used a condom during their last sexual encounter compared to those who did not have multiple sexual partners.

**Table 3.** Bivariate logistic regression analysis of selected background and socio-economic characteristics of men aged 50 years and above by their sexual behaviour.

	Condom use		Multiple sexual partners	
Variables	UOR	CI	UOR	CI
*Multiple sexual partners*				
No	1	–	n/a	–
Yes	6.29*	4.89–8.09	–	–
*HIV Prevention/transmission knowledge*				
Low	1	–	1	–
Medium	3.45*	1.72–6.94	1.51*	1.03–2.21
High	4.33*	2.17–8.65	1.56*	1.07–2.28
*Ever been tested for HIV*				
No	1	–	1	–
Yes	3.26*	2.57–4.12	1.82*	1.51–2.20
**Age**	1.04*	1.00–1.08	1.03*	1.00–1.06
*Place of residence*				
Urban	1	–	1	–
Rural	0.50*	0.40–0.63	0.66*	0.54–0.79
*Religion*				
Catholics	1	–	1	–
Other Christians	1.04	0.79–1.37	0.77*	0.62–0.96
Muslim	0.41*	0.29–0.58	0.29*	0.22–0.39
Other	1.23	0.84–1.79	0.81	0.59–1.11
**Age at 1st Intercourse**	1.01	0.99–1.04	0.95*	0.93–0.98
*Type of marriage*				
Monogamy	1	–	1	–
Polygamy	0.45*	0.31–0.65	0.95	0.73–1.24
*Education level*				
No education	1	–	1	–
Primary	3.98	2.67–5.92	2.23*	1.72–2.90
Secondary+	6.54*	4.35–9.83	3.17*	2.40–4.19
*Wealth index*				
Poor	1	–	1	–
Middle	1.81*	1.27–2.59	1.22	0.94–1.59
Rich	2.77*	2.07–3.69	1.53*	0.07–0.15

Significant at *p < .05.

Note: n/a, not applicable; UOR, unadjusted odds ratios; CI, confidence interval.

#### Multiple sexual partners

Results from the bivariate analysis of multiple sexual partners presented in Table 3 show that current age is positively associated with having multiple sexual partners (UOR 1.03; CI 1.00–1.06). Men with primary (UOR 2.23; CI 1.72–2.90) and secondary (UOR 3.17; CI 2.40–4.19) level of education were more likely to report multiple sexual partners compared to those men with no education. Men from rich households were (UOR 1.53; CI 0.07–0.15) more likely to engage in multiple sexual relationships compared to those from poor households. Compared to men who have never taken an HIV test, those who had taken an HIV test at any point in their lifetime were more likely to have multiple sexual partners (UOR 1.82; CI 1.51–2.20). Men whose HIV prevention and transmission knowledge score was medium (UOR 1.51; CI 1.03–2.21) and high (UOR 1.56; CI 1.07–2.28) were more likely to have multiple sexual partners compared to those with a low score. The odds of having multiple sexual partners among rural residents were lower compared to urban residents (UOR 0.66; CI 0.54–0.79). Other Christians (UOR 0.77; CI 0.62–0.96) and Muslim (UOR 0.29; CI 0.22–0.39) men had lower odds of having multiple sexual partners compared to Catholics. Age at first sex (UOR 0.95; CI 0.93–0.98) was also significantly associated with having multiple sexual partners.

### Results from the multivariate analysis

#### Condom use

In Table 4 the multivariate model for condom use shows that age at first sex, level of education, multiple sexual partners and ever been tested for HIV were significant predictors of condom use among older men in SSA region. Men who initiated sex at a later age were more likely to use a condom at last sex compared to those who started having sexual intercourse at an earlier age (adjusted odds ratio (AOR) 1.05; CI 1.02–1.08). Compared to men who had no education, those with primary education (AOR 4.47; CI 2.27–8.80) and secondary plus education (AOR 5.95; CI 2.94–12.05) were more likely to have used a condom during their last sex. Men who reported multiple sexual partners (AOR 4.33; CI 3.11–6.03) were more likely to have used a condom at last sex compared to those who did not have multiple sexual partners. Men who had taken at least one HIV test in their lifetime (AOR 2.01; CI 1.51–2.70) were more likely to use a condom during sexual intercourse compared to those who had never taken an HIV test in their lifetime.

**Table 4.** Multivariate logistic regression analysis assessing the relationship between all the selected characteristics and sexual behaviour in SSA.

	Condom use		Multiple sexual partners	
Variables	AOR	CI	AOR	CI
*Multiple sexual partners*				
No	1	–	n/a	–
Yes	4.33*	3.11–6.03	–	–
*HIV prevention/transmission knowledge*				
Low	1	–	1	–
Medium	2.02	0.77–5.27	1.22	0.69–2.16
High	1.85	0.71–4.83	1.10	0.62–1.95
*Ever been tested for HIV*				
No	1	–	1	–
Yes	2.02*	1.51–2.70	1.41*	1.11–1.81
**Age**	0.97	0.92–1.02	1.04*	1.00–1.10
*Place of residence*				
Urban	1	–	1	–
Rural	0.89	0.64–1.25	0.72*	0.54–0.97
*Religion*				
Catholics	1	–	1	–
Other Christians	1.07	0.67–1.73	0.75*	0.57–0.98
Muslim	1.54	0.98–2.48	0.46*	0.31–0.69
Other	1.56	0.98–2.48	0.65*	0.42–0.99
**Age at 1st Intercourse**	1.05**	1.02–1.08	0.97*	0.94–1.00
*Type of marriage*				
Monogamy	1	–	1	–
Polygamy	0.68	0.45–1.04	1.35	0.99–1.82
*Education level*				
No education	1	–	1	–
Primary	4.48***	2.28–8.81	1.34	0.92–1.94
Secondary+	5.95***	2.94–12.05	1.82*	1.20–2.77
*Wealth index*				
Poor	1	–	1	–
Middle	1.31	0.84–2.04	1.05	0.74–1.47
Rich	1.16	0.75–1.79	0.79	0.56–1.12

Significant at *p < .05.

Note: n/a, not applicable; AOR, adjusted odds ratio; CI, confidence interval.

#### Multiple sexual partners

In Table 4 the multivariate model for multiple sexual partners show that ever been tested for HIV, current age, place of residence, religion and education level are significant predictors of multiple sexual partners among older men in SSA. Compared to men who had never taken an HIV test, men who had ever been tested for HIV were more likely to have multiple sexual partners (AOR 1.41; CI 1.11–1.81). The likelihood of having multiple sexual partners increased with age (AOR 1.04; CI 1.00–1.10). Rural residents (AOR 0.72; CI 0.54–0.97) were less likely to have multiple sexual partners compared to urban residents. Men affiliated to other religions (AOR 0.65; CI 0.42–0.99), other Christians (AOR 0.75; CI 0.57–0.98) and Muslims (AOR 0.46; CI 0.31–0.69), had lower odds of having multiple sexual partners compared to Catholics. Men with secondary or higher level of education (AOR 1.82; CI 1.20–2.77) were more likely to have multiple sexual partners compared to men who had no education.

## Discussion

This study examined the covariates of high-risk sexual behaviour among men aged 50 years and above in SSA. The study showed that a larger proportion of men aged 50 years plus within this region were sexually active within four weeks prior to the survey. Similar findings have been reported in other studies (Cooperman et al., 2007; Freeman & Anglewicz, 2012; Knodel & Chayovan, 2001; Peltzer et al., 2010; Rosenberg et al., 2017; Todd et al., 2009). Peltzer et al. (2010) study among older South Africans aged 50 years plus showed that 41.1% of the study population were sexually active in the past 12 months; Freeman and Anglewicz (2012) reported that 83.8% of men in Malawi aged between 50 and 64 years had sex in the past year. This shows that sexual activity remains an important aspect of the well-being of individuals throughout their life cycle. Therefore, older men are also at risk of engaging in activities that transmit HIV since they are sexually active and should be targeted by prevention programmes. The call for urgent need for HIV prevention programmes targeted at older populations (50 years plus) has also been highlighted in other studies that have been conducted within the SSA region (Freeman & Anglewicz, 2012; Rosenberg et al., 2017), Africa as a whole (Negin et al., 2012) as well as in developed countries (Negin, Rozea, & Martiniuk, 2014).

Moreover, a substantial proportion of men aged 50 years and above within this region tend to engage in unsafe sexual behaviours such as having multiple sexual partners and unprotected sex, as shown by low condom use at last sex. This finding is consistent with what has been found in other studies (Kimuna & Djamba, 2005; Kinga et al., 2010; Kongnyuy, Wiysonge, Mbu, Nana, & Kouam, 2006; Oyediran et al., 2010; Uchudi, Magadi, & Mostazir, 2010). Kinga et al. (2010) study among elderly Nigerians aged 65 years and above showed that 30% of the sample engaged in extramarital relationships and only 7.3% reported condom use. Unsafe sexual behaviours such as having multiple sexual partners and unprotected sex are the main pathways through which HIV infection and other sexually transmitted infections are spreading within the SSA region (Mah & Halperin, 2010; Uchudi et al., 2010), therefore older men are a vulnerable age group.

The level of accurate HIV-related knowledge and uptake of the HIV test among older men, 50 years and above is still fairly low. According to the results of this study, less than half of the study population had a score of high HIV knowledge and the percentage of those who had ever taken an HIV test was as low as 34%. This finding confirms what Negin et al. (2012) found in their study in nine cities in Africa that showed that people older than 50 years had lower levels of HIV-related knowledge and were less likely to have ever been tested for HIV compared to those aged between 25–49 years. Low levels of accurate knowledge on HIV prevention among men aged 50 years and above indicate that this age group is not fully equipped to make informed decisions about their own sexual behaviour and also their ability to advise those who are under their care and provide guidance is limited. This contributes to increased HIV risk. Therefore, there is a need for programmes aimed at raising HIV awareness, knowledge and uptake of HIV testing among older men as this will not only benefit the older population but also the younger generation. Older people aged 50 years and above are usually the main caregivers of those affected or infected by HIV in the SSA region (Ssengonzi, 2007).

Men who had multiple sexual partners, primary or secondary plus education and those who had ever been tested for HIV were more likely to use a condom at last sex compared to their counterparts. Oyediran et al. (2010) study in Nigeria also showed high-level condom use among men who reported multiple sexual partners. The authors argued that this result suggested that men who engage in such high-risk sexual behaviour may be aware of the risk of contracting sexually transmitted infections, therefore, take precautions (Oyediran et al., 2010). However, the effectiveness depends on the correctness and consistence of condom use which needs to be probed further.

The practice of protective sex among those with higher levels of education has also been documented in other studies (Ahmed et al., 2001; Lagarde et al., 2001). Lagarde et al. (2001) study showed that educated men tend to practice protective sex as level of education was found to be a predictor of condom use with non-spousal partners in the selected cities in SSA. Sormanti and Shibusawa (2007) study showed that high-risk sexual behaviours among the older population were significantly more common among those with low educational levels. The study of Kongnyuy et al. (2006) among Cameroonian men showed contrary results, where unsafe sexual behaviours were found to be common among those men with education compared to those with no education (Kongnyuy et al., 2006)*.* The discrepancies in findings of Kongnyuy et al. (2006) with the results of our study could be due to different DHS rounds that were used for each study as well as differences in study samples, Kongnyuy et al. (2006) sample was made up of men aged between 15 and 59 years whilst in this study we only considered those aged 50 years and above.

Ever been tested for HIV was also found to be a predictor of condom use in SSA region. The explanation for the practice of safe sex among older men who had ever taken an HIV test could be that they were aware of their HIV status and their use of a condom during sex was a protective measure to prevent being infected or infecting others. It could also be a sign that they were knowledgeable about the importance of practicing safe sex which is usually emphasized during voluntary HIV counselling and testing (Fonner, Denison, Kennedy, O’Reilly, & Sweat, 2012).

Initiating sexual intercourse at an older age was also found to be significantly associated with condom use. This is consistent with several studies where risky sexual behaviours were found to be common among those who initiated sex at an early age compared to those who started having sex at an older age (Harrison, Cleland, Gouws, & Frohlich, 2005; Kaplan, Jones, Olson, & Yunzal-Butler, 2013; Li et al., 2015).

We also found that high-risk sexual behaviour such as having multiple sexual partners was significantly more common among men who had ever taken an HIV test, older men and those men who had secondary or higher levels of education. Oyediran et al. (2010) reported similar findings that men who had ever taken an HIV test were more likely to engage in extramarital relationships. Oyediran et al. (2010) pointed out that this could be a problem of reverse causation where men who engage in extramarital relationships are prompted to take an HIV test because of their higher exposure to the risk of infection.

The relationship between multiple sexual partners and secondary or higher levels of education has also been documented in other studies (Kongnyuy et al., 2006; Uchudi, Magadi, & Mostazir, 2012). This finding shows that education does not necessarily reduce the likelihood of involvement in high-risk sexual behaviour such as having multiple sexual partners. This confirms Uchudi et al. (2012) findings on determinants of multiple sexual partners in SSA. However, Kimuna and Djamba (2005) found no association between education and multiple sexual partners among Zambian men.

Multiple sexual partners were significantly less common among rural residents compared to urban residents. Similar findings have been found among Cameroonian men (Kongnyuy et al., 2006), Tanzanian adults in Arusha region (Mnyika, Klepp, Kvale, & Ole-King’Ori, 1997) and in SSA region (Uchudi et al., 2012) where risky sexual behaviours (multiple sexual partners) have been found to be more prevalent among urban residents compared to rural residents.

The positive association between age and multiple sexual partners’ result is contrary to findings that have been found in other studies, where the likelihood of having multiple sexual partners reduced as age increased (Kimuna & Djamba, 2005; Uchudi et al., 2012).

We also found that Catholic men were more likely to have multiple sexual partners compared to men who were affiliated to other religions (other Christians, Muslims and other) and this confirms findings from other studies (Adamczyk & Hayes, 2012; Hill et al., 2004). It is argued that the extent to which religion can influence individual lives depends on the incorporation and individual’s commitment to the doctrines and policies of their respective religious affiliations (Garner, 2000; Lehrer, 2004). Commitment is said to be more important than religious affiliation in affecting sexual attitudes and behaviour (Odimegwu, 2005; Smith, 1998).

## Limitations of the study

The results of this study should be interpreted with caution due to certain limitations. First, given the cross-sectional nature of the DHS data that was used causality between the compared variables cannot be determined. Second, we used multiple sexual partners as a proxy for high risky sexual behaviour but we did not take into consideration the timing of relationships (concurrent/serial or not) due to data limitations. The timing of relationships is an important issue in determining the extent of the risk.

## Conclusion

In conclusion, we found that older men aged 50 years and above in SSA are sexually active. Also, a significant proportion of them tend to engage in high-risk sexual behaviours such as having multiple concurrent sexual partners and unprotected sex which put them at risk of HIV infection. Involvement in high risk sexual behaviour also varies with personal and household attributes and the sexual behaviour being investigated. Therefore, there is a need for HIV prevention and intervention programmes that are tailor-made to meet the needs of older people. Also, there is an urgent need for inclusion of those older than 50 years in national surveys that collect information on sexual behaviour, to promote more comprehensive research on sexual behaviour of elderly people. This will generate more knowledge that can be incorporated in the development of prevention and intervention programmes that promote safe sexual behaviours within this age group.
